# Supplementary material for: An Exploration of Dynamic Changes in the Mulberry Growth Process Based on UPLC-Q-Orbitrap-MS, HS-SPME-GC-MS, and HS-GC-IMS
Source: Foods. 2023 Sep 6;12(18):3335. doi: 10.3390/foods12183335 (PMC10529768; doi:10.3390/foods12183335)
Supplement: Supplementary file 1 [file foods-12-03335-s001.zip › foods-2530546-supplementary.pdf]

Supplementary materials

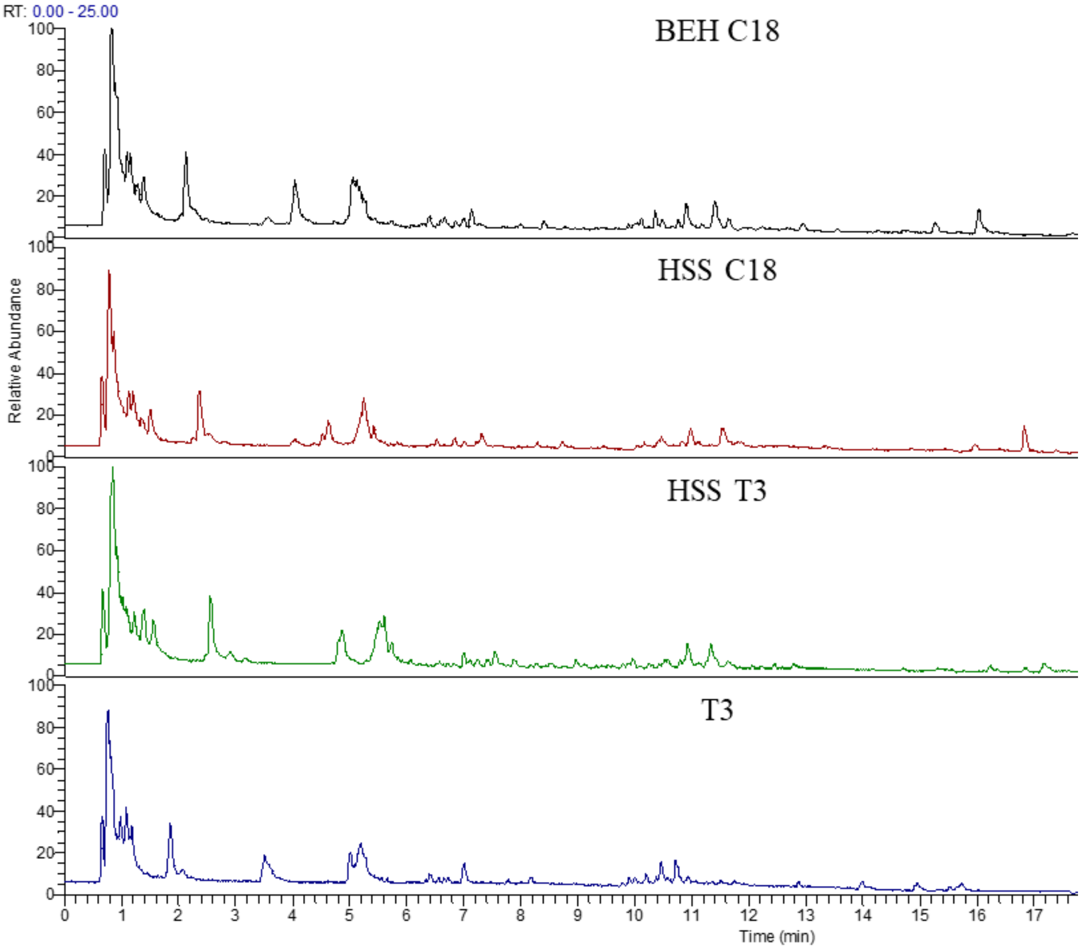

Figure S1. TIC of different chromatographic columns

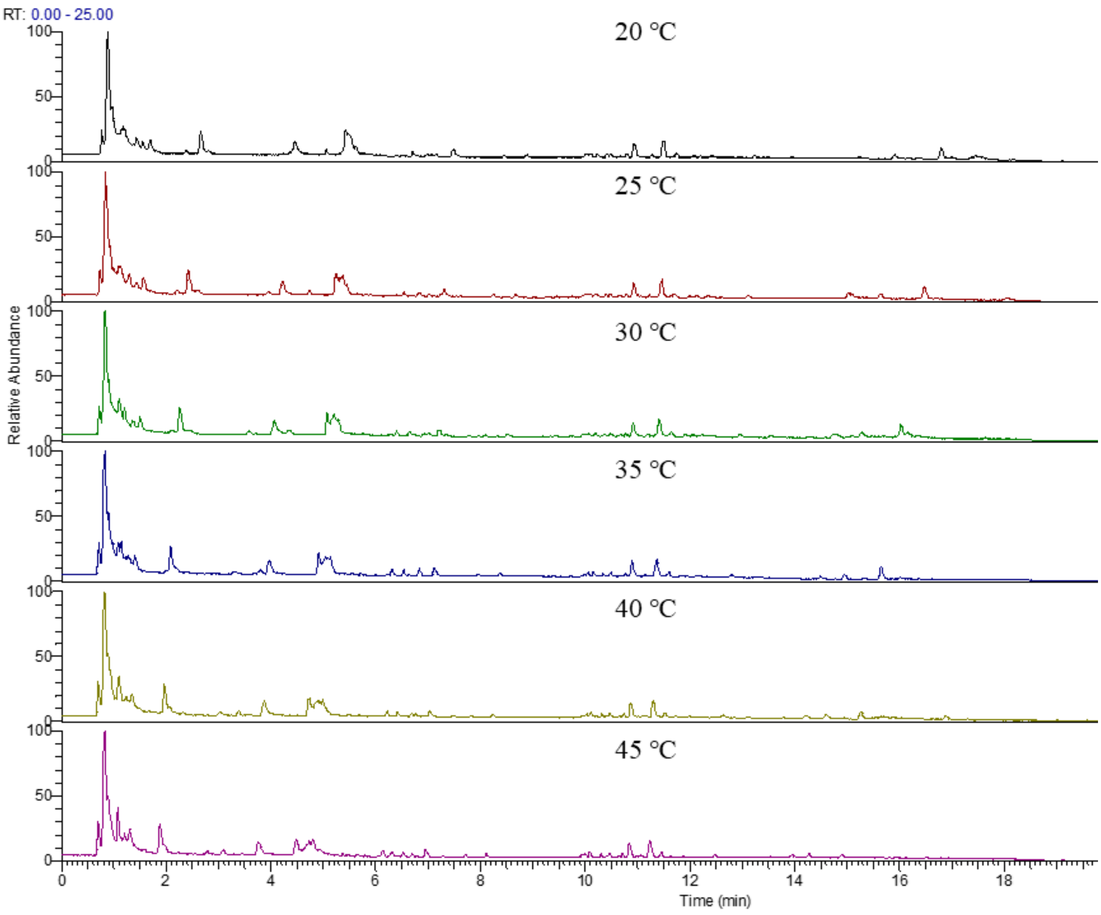

Figure S2. TIC of different column temperatures

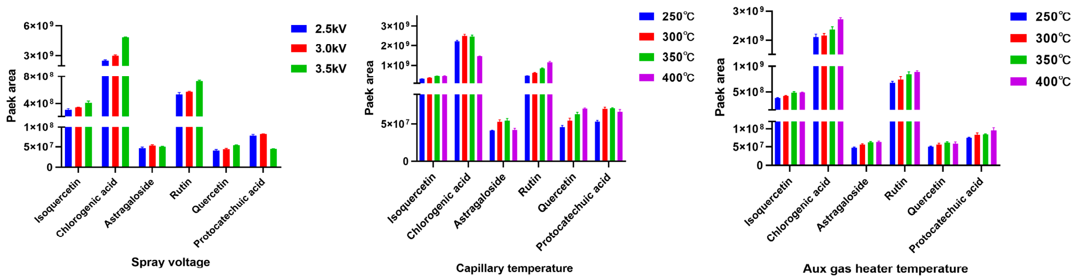

Figure S3. Optimization of Q-Orbitrap-MS conditions

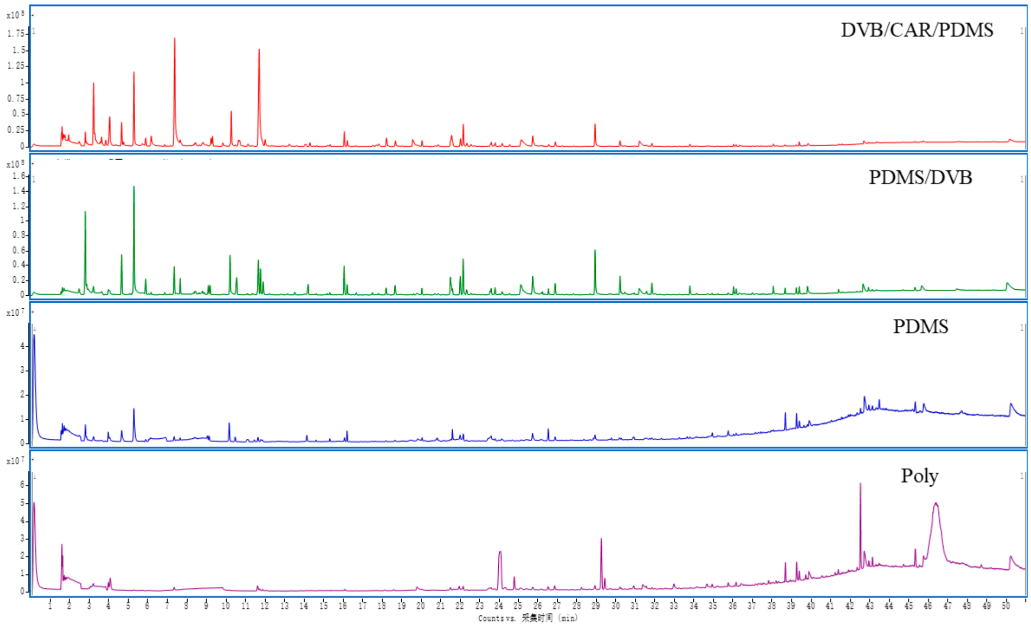

Figure S4. Optimization of extraction fiber head

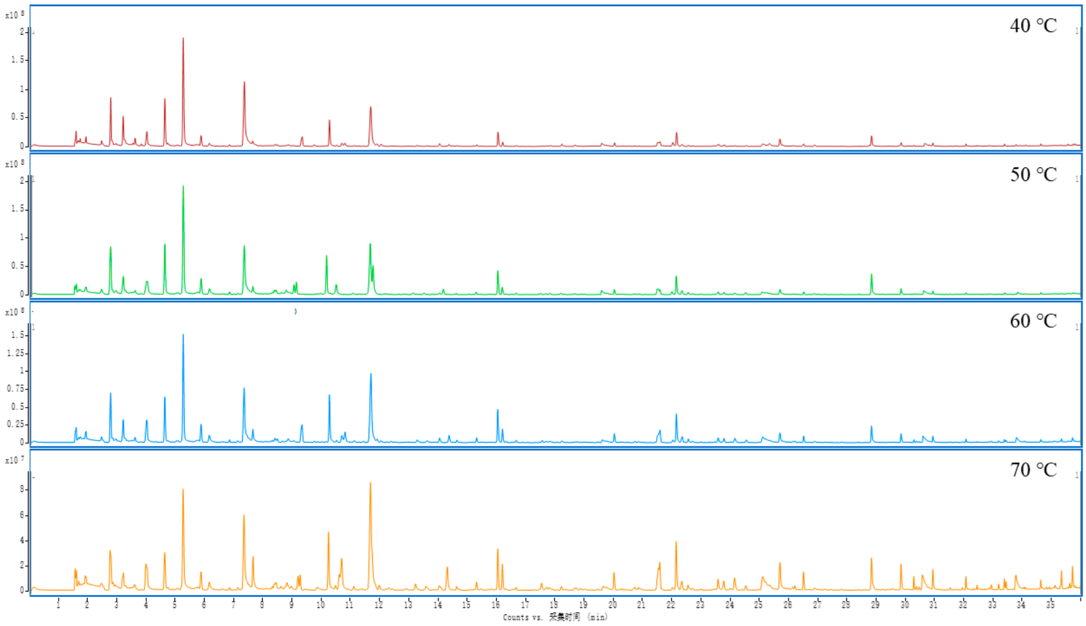

Figure S5. Optimization of incubation temperature

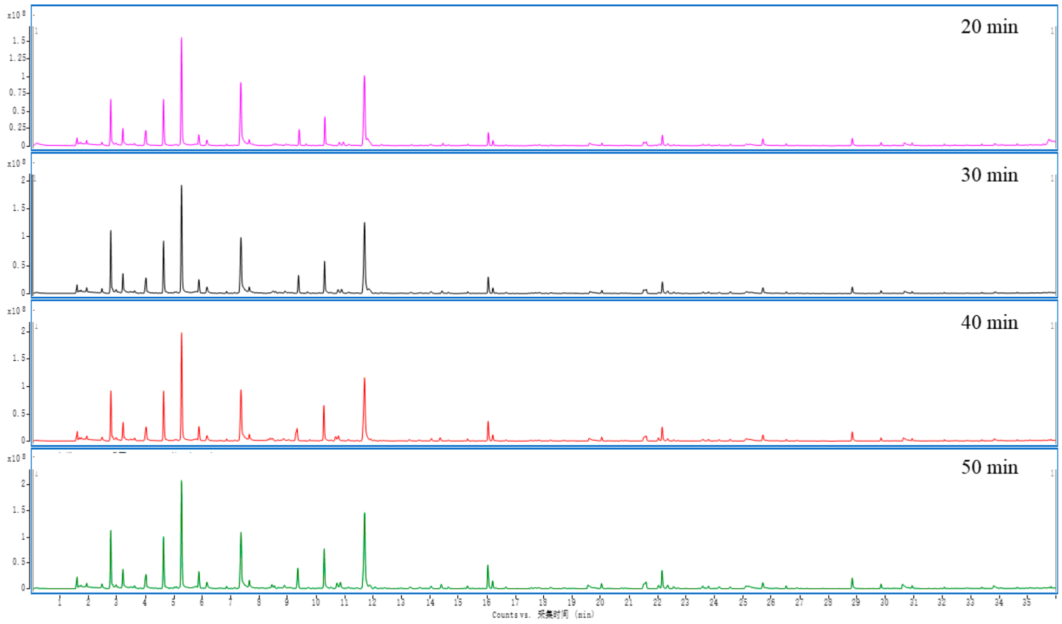

Figure S6. Optimization of extraction time

Table S1 results of absorbance and DPPH radical clearance rate

Stage	Concentration (mg/mL)	A_1-1	A_1-2	A_1-3	A_2-1	A_2-2	A_2-3	A_3-1	A_3-2	A_3-3	R1 (%)	R2 (%)	R3 (%)
Stage I	0.1	0.835	0.847	0.835	0.001	0.001	0.001	1.042	1.097	1.105	19.96	23.97	24.52
	0.2	0.738	0.747	0.734	0.001	0.001	0.001				29.27	32.82	33.67
	0.4	0.577	0.551	0.557	0.001	0.002	0.001				44.72	47.58	49.68
	0.6	0.413	0.407	0.428	0.001	0.002	0.001				60.46	62.53	61.36
	0.8	0.278	0.296	0.319	0.002	0.002	0.002				73.51	74.84	71.31
	1	0.174	0.207	0.202	0.002	0.002	0.003				83.49	84.32	81.99
	1.5	0.117	0.104	0.105	0.003	0.003	0.002				89.06	89.61	90.68
	2	0.11	0.094	0.093	0.003	0.004	0.003				89.73	90.34	91.86
	2.5	0.093	0.087	0.091	0.004	0.005	0.004				91.46	91.98	92.13
Stage II	0.1	0.821	0.814	0.804	0.001	0.001	0.001	1.042	1.097	1.105	21.31	25.25	27.33
	0.2	0.753	0.739	0.73	0.001	0.001	0.001				27.83	31.45	34.03
	0.4	0.614	0.598	0.582	0.001	0.003	0.001				41.17	44.30	47.42
	0.6	0.482	0.473	0.469	0.001	0.002	0.001				53.84	56.24	57.65
	0.8	0.378	0.37	0.363	0.001	0.002	0.002				63.82	65.72	67.33
	1	0.295	0.288	0.276	0.002	0.002	0.002				71.88	73.29	75.20
	1.5	0.136	0.121	0.117	0.002	0.003	0.002				87.14	87.88	89.59
	2	0.09	0.091	0.088	0.002	0.003	0.003				91.55	92.07	92.31
	2.5	0.083	0.084	0.08	0.003	0.004	0.004				92.32	92.80	93.12
Stage III	0.1	0.749	0.757	0.744	0.000	0.000	0.001	1.042	1.097	1.105	28.12	31.72	32.76

	0.2	0.678	0.663	0.656	0.000	0.000	0.001				34.93	38.20	40.72
	0.4	0.549	0.548	0.569	0.001	0.001	0.001				47.41	50.05	48.60
	0.6	0.443	0.427	0.445	0.002	0.002	0.002				57.68	59.80	59.91
	0.8	0.343	0.368	0.362	0.002	0.002	0.002				67.27	68.92	67.42
	1	0.247	0.282	0.235	0.003	0.004	0.003				76.58	77.85	79.00
	1.5	0.114	0.194	0.095	0.004	0.005	0.005				89.44	90.06	91.86
	2	0.089	0.101	0.085	0.006	0.006	0.005				92.03	92.43	92.76
	2.5	0.083	0.084	0.076	0.008	0.008	0.007				92.80	93.16	93.76
	0.1	0.781	0.777	0.764	0.003	0.003	0.003				25.34	29.08	31.13
	0.2	0.643	0.643	0.623	0.006	0.006	0.007				38.87	41.93	44.25
	0.4	0.365	0.376	0.386	0.013	0.014	0.013				66.22	68.00	66.24
	0.6	0.203	0.218	0.165	0.022	0.021	0.021				82.63	83.41	86.97
Stage IV	0.8	0.184	0.216	0.157	0.028	0.028	0.028	1.042	1.097	1.105	85.03	85.78	88.33
	1	0.181	0.194	0.156	0.033	0.034	0.034				85.80	86.60	88.96
	1.5	0.178	0.189	0.15	0.051	0.052	0.052				87.81	88.51	91.13
	2	0.191	0.202	0.154	0.068	0.071	0.065				88.20	89.06	91.95
	2.5	0.202	0.218	0.163	0.085	0.088	0.083				88.77	89.61	92.76

A_1: the absorbance value of the sample after reaction with DPPH; A_2: the absorbance value of the sample after reaction with anhydrous ethanol; A_3: The absorbance value after the reaction between DPPH and anhydrous ethanol; R: free radical clearance rate of DPPH.
